# In Situ Generation of Gel Foam via Heat‐Accelerated Calcium Ion Release From Nano‐CaCO_3_ for Lithium‐Ion Battery Fire Suppression and Prevention

**DOI:** 10.1002/advs.77882

**Published:** 2026-09-27

**Authors:** Xiaoyang Yu, Cuiping Li, Mingjun Xu, Man Pun Wan

**Affiliations:** ^1^ State Key Laboratory of Fire Science University of Science and Technology of China Hefei China; ^2^ School of Mechanical and Aerospace Engineering Nanyang Technological University Singapore Singapore

**Keywords:** battery fire, fire fighting, gel foam, stimulus‐responsive materials, thermal runaway propagation

## Abstract

Thermal runaway and its propagation in lithium‐ion battery packs pose significant safety risks to energy storage systems. While complete immersion in water‐based agents is an effective suppression strategy, conventional agents often fail to achieve sustained battery encapsulation because of low viscosity and rapid runoff. In this study, we report a thermally accelerated in situ crosslinking strategy to create an alginate gel foam for excellent battery fire suppression and propagation prevention. Our approach utilizes nano‐CaCO_3_ as a dormant calcium ion source and δ‐gluconolactone (GDL) as a slow acidifier. At ambient temperature, the extremely low solubility of nano‐CaCO_3_ results in slow Ca^2+^ release in aqueous solution, with the mixture maintaining low viscosity and excellent foamability. After exposure to battery fire, GDL hydrolysis is accelerated, causing rapid nano‐CaCO_3_ dissociation, Ca^2+^ release, and, subsequently, rapid ionic crosslinking of the alginate, which transforms the aqueous foam into a stable, adhesive gel foam. In full‐scale tests, we determined that this gel foam extinguishes single‐cell fires rapidly and significantly inhibits thermal runaway propagation (TRP) in multi‐cell modules, outperforming water mist, water spray, and commercial aqueous film‐forming foam (AFFF). This stimulus‐responsive alginate gel foam offers a promising strategy for increasing the safety of lithium‐ion batteries.

## Graphical abstract

We present a heat‐accelerated in situ crosslinking strategy to create an alginate gel foam for lithium‐ion battery fire suppression. At ambient temperature, the formulation maintains low viscosity for easy foam generation. After exposure to fire, δ‐gluconolactone hydrolysis accelerates, enabling rapid Ca^2+^ release from CaCO_3_ and rapid ionic crosslinking of alginate. This process transforms the flowing foam into the gel foam, achieving complete battery encapsulation and cooling.

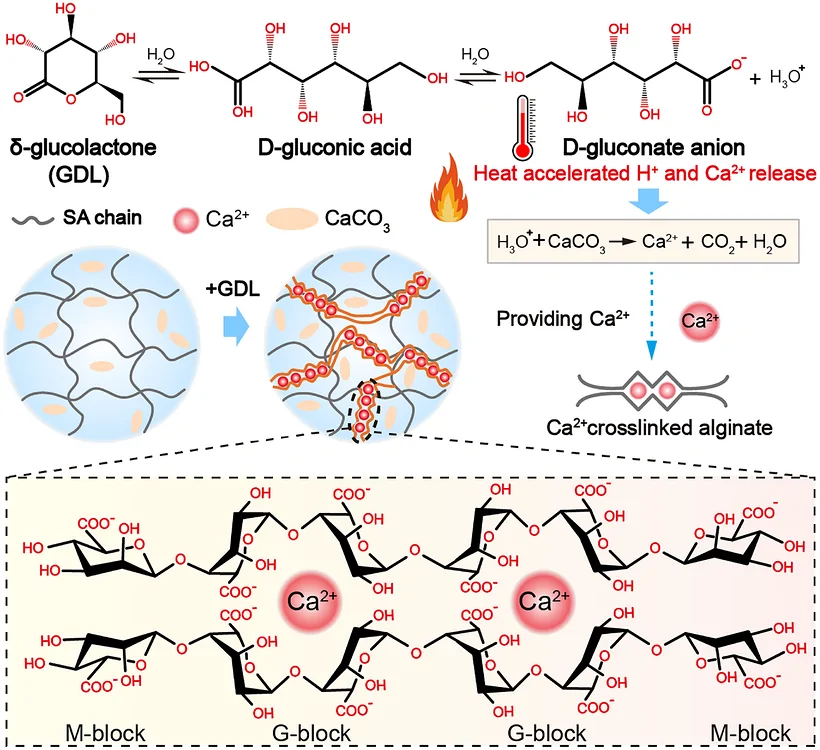

## 1 Introduction

With the advancement of electric vehicles and portable devices, lithium‐ion batteries are powering our lives. Their main advantages are high energy density, long cycle life, and environmental friendliness. However, lithium‐ion batteries are prone to thermal runaway, a process that often involves chemical reactions and can ultimately result in fire [1, 2, 3]. Lithium‐ion battery fires are characterized by high heat release rates and a prolonged duration of high temperatures [4, 5, 6]. Existing strategies, such as optimizing battery materials, improving thermal management systems, and upgrading flame‐retardant housings and separators, primarily focus on preventing the initiation of thermal runaway [7, 8, 9, 10, 11]. While crucial, these preventive measures cannot guarantee absolute safety, creating a critical need for active suppression technologies as a last line of defense [7, 8, 9, 10, 11]. Without such active protection, fire can quickly propagate from a single pack to adjacent battery packs. Therefore, effective suppression strategies are essential for restricting thermal runaway propagation and minimizing the consequences of battery fire incidents.

Fire suppression agents can inhibit combustion through heat absorption and oxygen isolation [12, 13]. They are generally sprayed onto the surface of the overheating battery through customized pipes or nozzles. For lithium‐ion battery fires, complete immersion using water‐based extinguishing agents, primarily water and aqueous foam, is considered an effective approach to suppress fire and prevent fire propagation [12]. Ideally, the battery pack experiencing thermal runaway would be fully enveloped in a “liquid pool” formed by the water, keeping adjacent cells below their self‐accelerated decomposition temperature and significantly inhibiting the spread of combustion and fire [12]. However, conventional water‐based agents have difficulty achieving complete and sustained battery submersion because of their low viscosity and tendency to run off [14], preventing effective encapsulation of the battery module.

The gelation of water‐based extinguishing agents can effectively reduce water evaporation and agent loss while improving thermal stability [15]. Compared with water, aqueous foam is more suitable for gelation strategies because foam is a yield‐stress fluid that behaves as a solid until a critical stress, known as yield stress, is applied, after which it flows like a liquid [16]. Ionic crosslinking is a typical method for inducing foam gelation and improving the yield stress of foam [17, 18]. Conventional approaches involve dripping a crosslinking agent (e.g., CaCl_2_) into a foaming solution containing sodium alginate (SA) or xanthan gum [19]. Ca^2+^ reacts with functional groups such as carboxyl groups in SA, leading to gelation in the liquid phase. However, gel formation occurs too rapidly (within a dozen seconds), resulting in high resistance to foaming and difficulty in mixing liquid with air in mechanical devices to generate sufficient foam [20, 21, 22, 23]. An ideal strategy should first achieve a formulation with low viscosity and high foamability. This formulation must then be capable of rapid in situ gelation triggered by contact with a lithium battery flame, thereby ensuring complete battery encapsulation. Although many studies have investigated the use of water or aqueous foam for the fire suppression of lithium batteries [12, 13, 14], reports on in situ heat‐accelerated gelation for fire suppression remain scarce.

**SCHEME 1:**
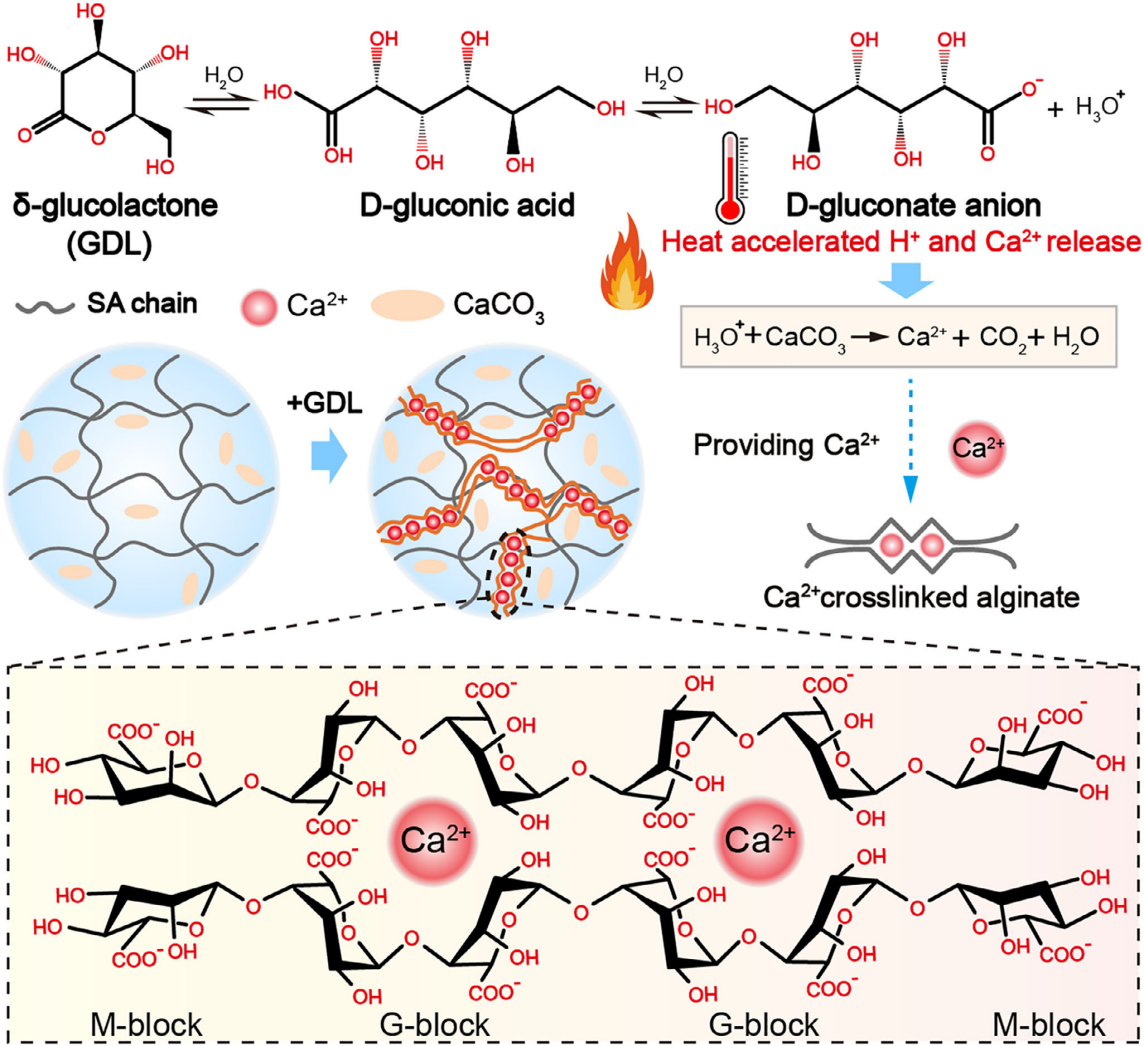
Controlled ionic crosslinking strategy involving three steps: the hydrolysis of GDL to generate H^+^, the dissociation of CaCO_3_ to release Ca^2+^, and the crosslinking of SA chains by Ca^2+^. The high temperature generated by the flame can accelerate the hydrolysis of the GDL and the release of H^+^.

This study proposes a novel thermally accelerated in situ crosslinking method to create alginate gel foam for battery fire suppression. In contrast to previous studies, the gelation (also referred to as the cross‐linking process) of foam is induced by the heat released from the flames during thermal runaway of the battery. Nano‐CaCO_3_ is used as the Ca^2+^ source. The extremely low solubility of nano‐CaCO_3_ results in slow Ca^2+^ release in aqueous solution. GDL undergoes hydrolysis in water to slowly release H+ ions. These protons then react with nano‐CaCO_3_, inducing the slow release of Ca^2+^ (Scheme 1). At room temperature, Ca^2+^ release from nano‐CaCO_3_/GDL mixtures is very slow, allowing the liquid containing SA and surfactant to achieve low viscosity and high foamability. The high temperature generated by lithium battery thermal runaway facilitates GDL hydrolysis and accelerates Ca^2+^ release, enabling rapid ionic crosslinking of the foam around the overheating battery. This in situ‐formed gel foam, facilitated by flame‐accelerated Ca^2+^ release, significantly outperforms conventional fire extinguishing agents in suppressing and preventing lithium battery fires.

## 2 Results and Discussion

### 2.1 Stimuli‐Responsive Ionic Cross‐Linking and Mechanism

**FIGURE 1:**
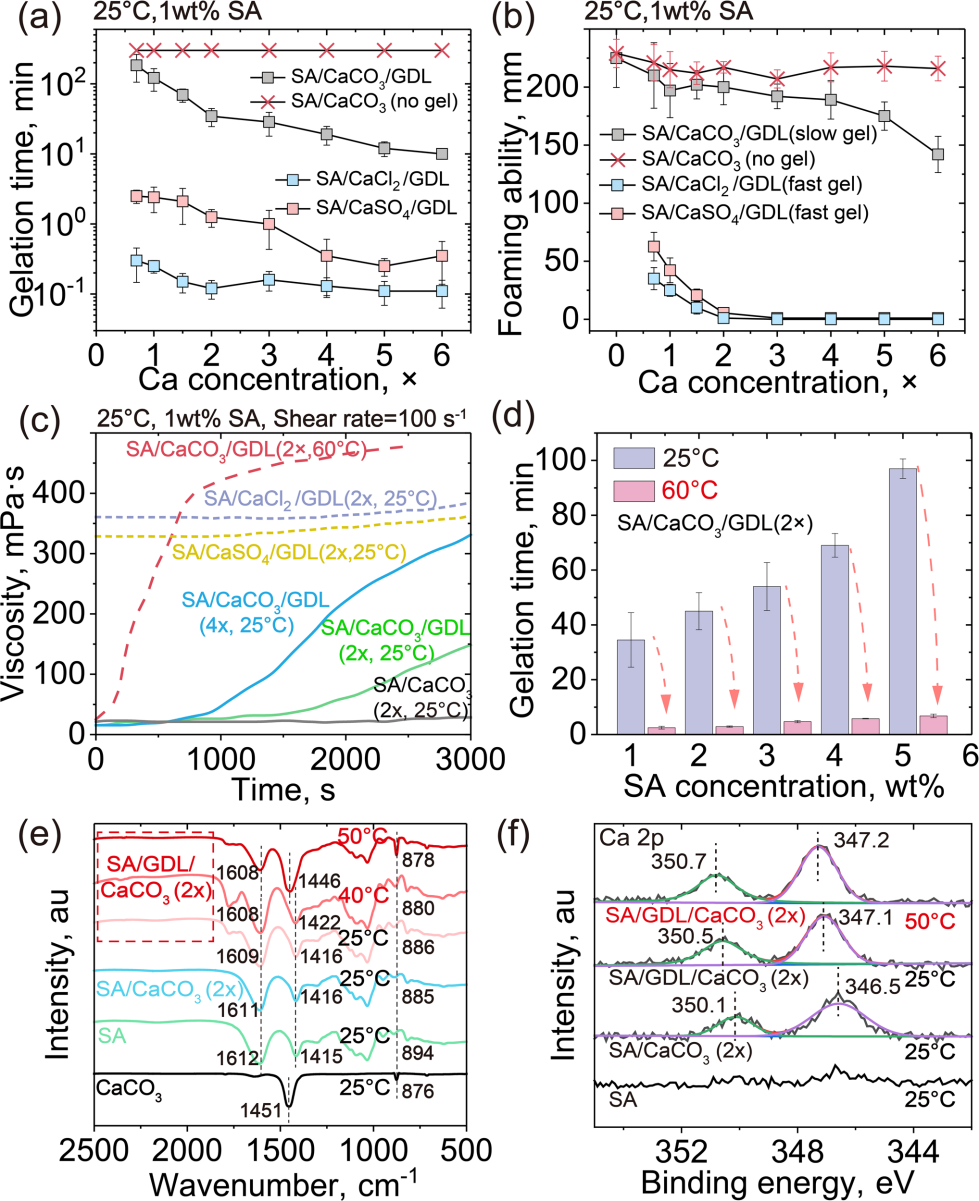
(a–b) Effects of Ca concentration on gelation time and foamability in the presence of three calcium sources with differing solubilities. (c) Viscosity versus time. (d) Effects of temperature on the gelation time of foam containing SA/CaCO_3_/GDL. (e–f) FTIR and Ca 2p XPS spectra for the freeze‐dried samples after heat treatment. The ER of the foam was 5.

SA is a linear copolymer composed of β–D‐mannuronic acid (M) and α‐L‐guluronic acid (G) residues linked by 1,4‐glycosidic bonds into homogeneous (MM and GG) and heterogeneous (MG and GM) blocks [24]. The SA molecular chains are rich in carboxyl groups, which can undergo ionic crosslinking with Ca^2+^ to rapidly form a 3D network gel. The influence of different Ca concentrations on gelation time in the presence of three calcium sources with differing solubilities (CaCO_3_<<CaSO_4_<<CaCl_2_) is shown in Figure 1a. Owing to the very low solubility of nano‐CaCO_3_ in water, incorporating nano‐CaCO_3_ into a 1 wt.% SA solution did not induce gelation within 300 min (Figure 1a). After GDL was added to the SA/CaCO_3_ mixture, the H^+^ released from the slow hydrolysis of GDL decomposed nano‐ CaCO_3_, releasing Ca^2+^. These ions subsequently participate in the crosslinking of the G‐blocks in the alginate chain (Scheme 1). The GDL/CaCO_3_ system releases Ca^2+^ at a slow rate, requiring tens to hundreds of minutes to create a complete gel (Figure 1a). This slow process results in a system with low viscosity, which is expected to readily facilitate foaming after the 1 wt.% APG is added. As a nonionic surfactant, alkyl polyglycoside (APG) is less likely to undergo ionic interactions or form precipitates in systems with relatively high concentrations of Ca^2+^, thus helping to maintain low surface tension (Figure S1) and the stability of the foam. In contrast, gelation between SA and either CaSO_4_ or CaCl_2_ occurred too rapidly (within 3 min).

Aqueous foam was produced by mixing a liquid and a gas in the modified Tessari method in the laboratory (Figure S2). However, when CaSO_4_ or CaCl_2_ is used as the calcium source, the SA solution is expected to undergo gelation prior to mixing with the gas. The foaming ability data in Figure 1b confirm this point. Using the standard Ross‒Miles method, we compared the foaming performance of freshly prepared solutions containing different calcium sources. Owing to the slower gelation initiation time, the SA/CaCO_3_/GDL formulation exhibited a high foaming capacity (initial foam height >180 mm) over a wide range of calcium concentrations, approaching that of the SA/CaCO_3_ formulation and exceeding that of the AFFF (Figure S3) and the commercial foam extinguishing agent formulation reported in our work [16, 25]. In contrast, formulations using CaSO_4_ or CaCl_2_ as the calcium source showed lower foaming capacity (<50 mm), even at lower calcium concentrations.

The viscosity changes over time for solutions containing different calcium sources at varying temperatures are shown in Figure 1c. At 25°C, the SA/CaCO_3_/GDL solution exhibited a high foaming capacity and a long gelation initiation time. Notably, when the temperature increased from 25°C to 60°C (Figure 1d), the gelation time of the SA/CaCO_3_/GDL solution decreased by an order of magnitude. On the one hand, higher temperatures increase the solubility of CaCO_3_. On the other hand, the ionic cross‐linking of Ca^2+^ and the SA chain is initiated by the hydrolysis of the GDL, and increasing the temperature can accelerate the hydrolysis of the GDL. The gelation rate decreased with increasing SA concentration (Figure 1d). The higher viscosity induced by higher SA concentrations, which increases interchain hindrance and friction, likely slows this rearrangement, impeding the formation of effective crosslinks. FTIR and XPS tests were employed to confirm the in situ Ca^2+^ cross‐linking of SA at different temperatures. As shown in Figure 1e, the peaks at 1415 and 1612 cm^−1^, attributed to the symmetric and asymmetric stretching vibrations of the COO^−^ groups in SA [19], are blueshifted to 1416 cm^−1^ and redshifted to 1609 cm^−1^, respectively. These shifts are attributed to the participation of SA in intermolecular crosslinking with Ca^2+^. Furthermore, an increase in the intensity of the characteristic peaks of CaCO_3_ (near 1451 and 876 cm^−1^) was observed when the temperature was raised from 25°C to 50°C, possibly associated with the hydrolysis of the GDL and the gradual release of Ca^2+^. The Ca 2p XPS spectrum of the GDL‐treated SA/CaCO_3_ mixture (Figure 1f) revealed an increase in binding energy compared with that of the untreated SA/CaCO_3_ sample (with peaks at 346.5 and 350.1 eV). This shift is characteristic of the cross‐linking between Ca^2+^ and the carboxylate groups of SA [26]. The uniform distribution of calcium on the surface of the freeze‐dried foam also confirmed the effectiveness of the cross‐linking (Figures S4 and S5). Moreover, the characteristic peak of the Ca 2p XPS spectrum shifts toward higher binding energy as the temperature increases, which further demonstrates that elevated temperatures accelerate the release of calcium ions and thereby promote cross‐linking between calcium and SA (Figure 1f).

**FIGURE 2:**
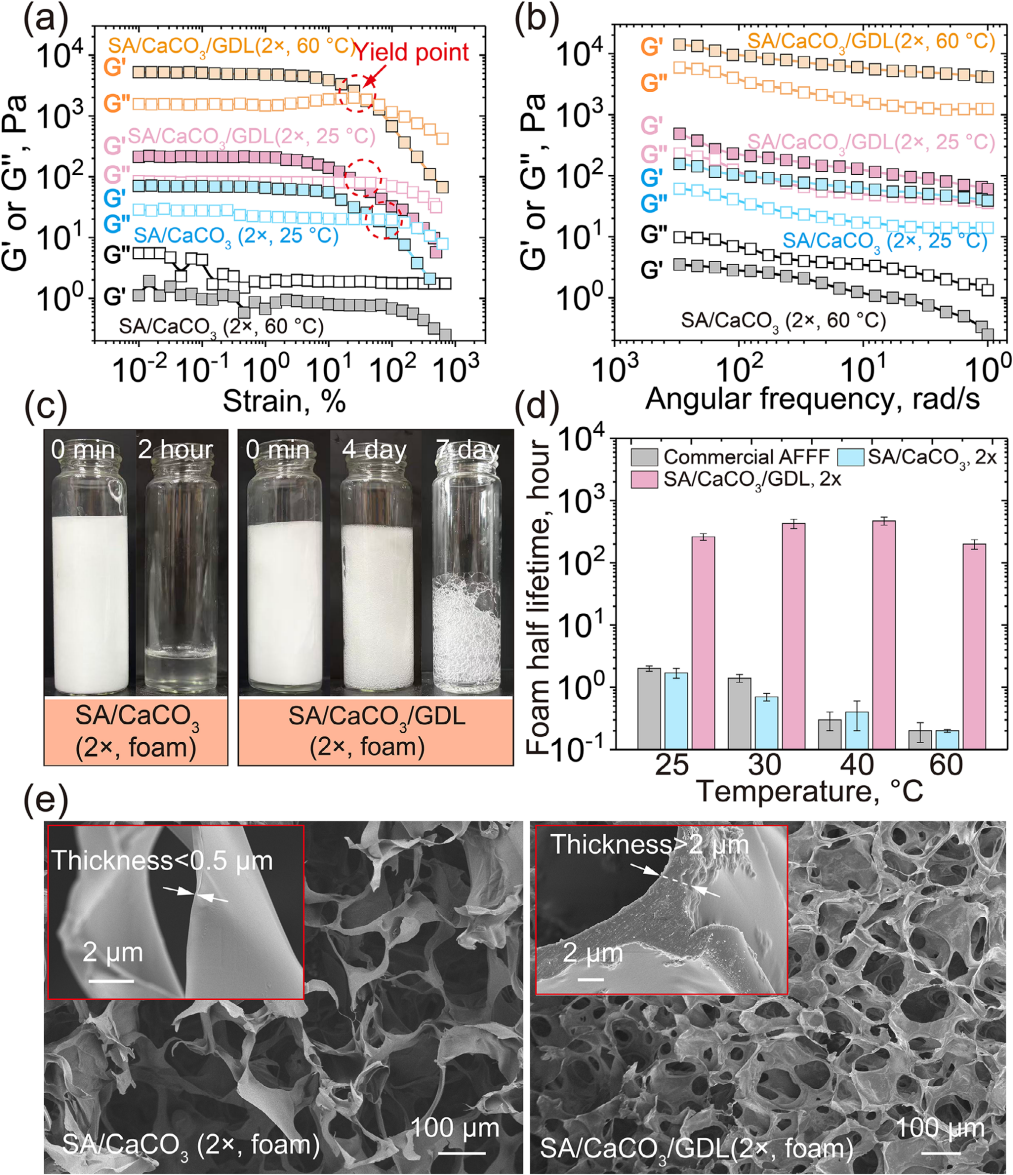
(a–b) Variations in G' and G'' of foam with strain and frequency for different formulations. (c) Photographs of the foam samples with and without added GDL after standing for different periods of time. (d) The half‐life of foams with different formulations. (e) Effects of the GDL on the microstructure of the SA foam (the Ca concentration is 2×). The ER of the foam was 5.

Rheological tests were performed on both foams prepared with 1 wt.% SA‐CaCO_3_‐GDL at 25°C and 60°C (Figure 2a,b). With respect to the foam with 1 wt.% SA‐CaCO_3_ (2×) at 60°C, oscillatory measurements (both amplitude and frequency sweeps) consistently revealed a G′ greater than the G', indicating liquid‐like, fluid behavior. At 25°C, a transition from solid‐like (G′>G″) to liquid‐like (G′<G″) behavior was observed with increasing strain. The crossover point of G' and G″ indicates irreversible deformation [27], allowing for the determination of yield stress. The SA/CaCO_3_ foam itself, a yield‐stress fluid, exhibited a low yield stress (<100 Pa). An increase in temperature reduces the yield stress of the SA/CaCO_3_ foam. For the sample containing the GDL, the increase in temperature induced gelation, and the yield stress increased by an order of magnitude (>1000 Pa), which enables the foam to be coated onto the lithium battery surface and effectively resist flow under gravity.

This unique gelation mechanism, which uses temperature to control the release of Ca^2+^, significantly enhances the stability of the foam. Unlike traditional surfactant‒polymer foams, which typically collapse within hours because of drainage and coarsening [28, 29], the in situ‐formed gel network effectively inhibits liquid drainage and bubble coarsening (Figure 2c, Figures S6 and S7). The qualitative analysis of foam stability is shown in Figure 2c,d. The foam produced by SA/CaCO_3_ degrades completely within a few hours. Remarkably, the gel foam produced with SA/CaCO_3_/GDL showed a long lifetime, which persisted for up to 200 h. Furthermore, this stability is maintained at higher temperatures. Even at 60°C, compared with the ∼10‐min half‐life of a commercial AFFF and the ∼5‐min half‐life of the SA‐CaCO_3_ foam without a GDL, the SA/CaCO_3_/GDL foam exhibited a half‐life of 100 h. This finding is attributed to accelerated Ca^2+^ release induced by the hydrolysis of GDL and interchain crosslinking of SA at high temperatures. SEM images reveal that gelation inhibits liquid drainage of foam and thickens the bubble films to more than 2 µm from <0.5 µm (Figure 2e), significantly improving the mechanical and thermal stability of the foam. Micro‐CT analysis of freeze‐dried foams also confirmed that SA/CaCO_3_/GDL foams have thicker lamellae and smaller pore sizes compared to SA/CaCO_3_ foams (Figures S8 and S9).

### 2.2 Lithium Battery Fire Extinguishing and TRP Inhibition Test

**FIGURE 3:**
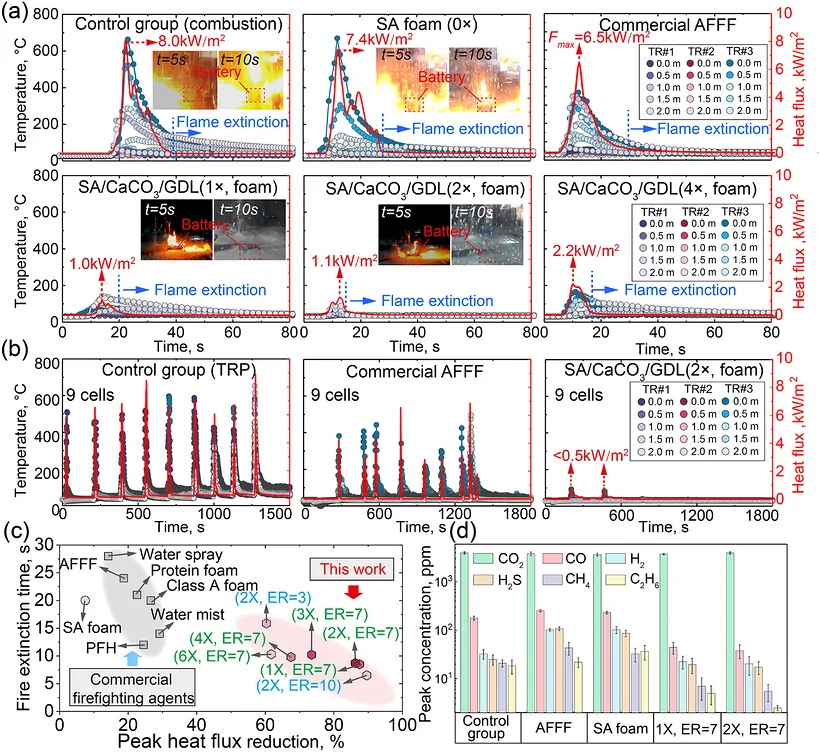
Temperature distribution inside the cabin during the (a) single‐cell fire‐fighting test and (b) TRP test of a 9‐cell module. (c) Comparison of firefighting performance between the gel foam used in this study and a commercial extinguishing agent. (d) Peak gas concentration inside the cabin during the single‐cell firefighting process.

Our tests were conducted in a real battery energy storage cabin [6] (Figure S10) on two scales: fire extinguishment on a single battery cell and suppression of TRP across a nine‐cell module. Figures S11–S16 present the thermal runaway parameter characterization of the battery we used, and the total heat release of a single battery reaches 2 MJ. The temperature distribution inside the cabin and on the surface of the battery during the fire suppression tests is shown in Figure 3 and Figure S17. Without any extinguishing agent, the single battery burned for 25 s, with a cabin temperature greater than 600°C and a peak heat flux (F_max_) of approximately 8 kW/m^2^. SA foam without a cross‐linking strategy and commercial AFFF showed no significant reduction in extinguishing time or F_max_. These foams are susceptible to degradation at high temperatures (Figure 2d) and have low yield stress (Figure 2a,b), preventing effective coverage and accumulation on the surface of thermal batteries. The thermally accelerated cross‐linked SA gel foam significantly reduced the combustion duration of the battery, with flames extinguishing within 8–10 s. At a 2× cross‐linking concentration, the cabin temperature decreased below 200°C, and the F_max_ was as low as 1.1 kW/m^2^. As the GDL induces the release of calcium ions from CaCO_3_ after heating, facilitating the cross‐linking of sodium alginate, the foam creates a highly adhesive and stable gel in situ after exposure to heat, enabling complete submersion and sustained cooling of the lithium battery (see the insert image in Figure 3a).

The effective suppression of thermal runaway in a 9‐cell lithium battery pack by the gel foam is shown in Figure 3b. During the continuous discharge of commercial AFFFs, all 9 cells experienced thermal runaway. In a 9‐cell lithium battery module, the average time interval for TRP between adjacent cells is approximately 100 to 200 s. In contrast, after the application of SA/CaCO_3_/GDL foam, only two cells experienced thermal runaway (Figure S17b), and the F_max_ remained less than 0.5 kW/m^2^. The gelation of the foam imparts high stability, enabling it to effectively block the jet fire and external thermal radiation from lithium‐ion batteries. Furthermore, compared with other commercial extinguishing agents, such as water mist, water spray, and perfluorohexanone (PFH), the SA/CaCO_3_/GDL foam resulted in faster extinguishing times and more efficient heat flux reduction (Figure 3c), particularly at high ER. The SA/CaCO_3_/GDL foam can block up to 50% to 90% of the radiant heat flux from the battery, whereas water and AFFFs can block only a maximum of 10% to 30% of the radiant heat flux. Moreover, compared with commercial AFFFs, thermally triggered gel foam rapidly envelops the battery surface and absorbs heat, leading to notable reductions in CO, H_2_, and H_2_S emissions from the battery during thermal runaway (Figure 3d). Its heat dissipation rate exceeds the heat transfer rate between the batteries (Figures S18–S21), ultimately blocking the propagation of thermal runaway.

### 2.3 Lithium Battery Fire Inhibition and Cooling Mechanism of Gel Foam

During thermal runaway, exothermic reactions within the battery release a substantial amount of heat, resulting in an internal temperature that is typically higher than the surface temperature. Driven by the temperature gradient, heat transfers from the interior to the surface of the battery and is subsequently dissipated into the surrounding environment through water or air. With respect to the thermally triggered SA/CaCO_3_/GDL gel foam, the successful suppression of the TRP is attributed to the heat dissipation rate from the battery surface to water and air (*q_c_*) exceeding the heat transfer rate between batteries (*q_h_*). In contrast, thermally unstable foams (such as AFFF and SA foam) exhibit poor thermal stability at high surface temperatures, where bubbles are prone to rupture, coarsen, and coalesce, and eventually break down into a water film that rapidly evaporates. Under these conditions, heat dissipation from the battery surface relies primarily on convection in air. Once *q_c_* falls below *q_h_*, the temperature gradient between the thermal runaway battery and adjacent batteries can trigger thermal runaway in neighbouring cells. Battery cooling relies primarily on air cooling and water cooling within the foam. The total heat dissipation can be regarded as the approximate sum of the heat removed by air and water (*Q_c_*).

(1) $$c_{p}m_{r}\Delta T=Q_{c}=Q_{\mathrm{air}}+Q_{w}$$

(2) $$q_{c}=Q_{c}/\Delta t=c_{p}m_{r}\Delta T/\Delta t$$

where *Q_air_* and *Q_w_* represent the heat removed by air and water, respectively. *m_r_* denotes the residual mass of the battery after thermal runaway. *c_p_* is the specific heat capacity of the battery (1.05 kJ/kg/°C, Figure S16), as determined by the accelerating calorimeter tests. *Δt* refers to the time interval during which the temperature decreases. *ΔT* and *q_c_* represent the temperature drop of the battery during this high‐temperature period and its average heat dissipation rate, respectively. We calculated *ΔT* in Figure S17 at all measurement points under the effect of the fire extinguishing agent and then used their average values to calculate *q_c_*.

We calculated the average heat dissipation rate *q_c_* under the influence of different fire extinguishing agents, as shown in Figures S18–S20. In the absence of a fire extinguishing agent, heat dissipation from the lithium battery surface after jet flame extinction relies primarily on convection in air. Owing to the low thermal conductivity of air, the temperature decrease of the battery is relatively slight. When foam is applied, heat dissipation occurs mainly through convection between the foam and the battery surface. However, sodium alginate SA foam exhibits poor thermal stability, degrading and draining rapidly upon contact with the hot battery surface. As a result, its heat dissipation capacity is limited—only approximately 90% higher than that in the no‐agent scenario. In contrast, compared with the SA foam, the thermally responsive SA/CaCO_3_/GDL foam demonstrates significantly superior heat dissipation performance, with a heat dissipation rate 346% higher than that in the no‐agent scenario. As the calcium ion concentration increases, the heat dissipation rate further increases, primarily because of the effective adhesion of the thermally cross‐linked foam to the battery surface. The optimal heat dissipation effect is achieved at a calcium‐to‐carboxyl molar ratio of 0.36 (2×), where the heat dissipation rate is 439% higher than that in the no‐agent scenario.

Increasing the ER enhances the air volume fraction within the foam, which accelerates foam accumulation and enables faster coverage of the lithium battery surface. At high ERs, the bubbles exhibit a polyhedral shape, whereas at lower ERs, they appear more spherical (Figure S19). The influence of ER on the thermal conductivity of commercial AFFF and SA/CaCO_3_/GDL gel foam is shown in Figure S21. At low ERs (with low air content), the thermal conductivities of commercial AFFF and SA/CaCO_3_/GDL foam differ little. As the ER increases, a separation between the two curves becomes evident (Figure S21): the thermal conductivity of the SA/CaCO_3_/GDL foam is greater than that of the AFFF, indicating enhanced heat transfer capability under comparable gas contents. The gel phase forms a continuous solid‐like heat conduction network and a thicker bubble film, which explains its higher thermal conductivity and enhanced cooling efficiency for batteries with identical gas contents. Additionally, the incorporation of nano‐CaCO_3_ is expected to contribute to the improved thermal conductivity of the foam. Although an increase in ER generally reduces the thermal conductivity of foam and thus its cooling efficiency, at ER = 7, the foam demonstrates optimal cooling performance (Figure S19). This is because a higher ER enhances the foam's yield stress [27], enabling more foam to remain stably adhered to the battery surface. In summary, the strategy proposed in this study, in which the fire‐extinguishing agent is gelled through thermally induced calcium ion release, can effectively mitigate the hazards associated with lithium battery thermal runaway. This is achieved primarily through highly efficient heat dissipation on the battery surface, with an average cooling powder (>1000 W) significantly higher than that of conventional water‐based extinguishing agents (300–600 W; Figure S20). Moreover, the thermal conductivity of the gel foam is greater than that of the commercial AFFF at the same gas content, enabling effective heat dissipation for lithium‐ion batteries.

## 3 Conclusions

In this study, we developed a novel, heat‐triggered in situ gelation strategy to create an alginate‐based gel foam for the effective suppression and prevention of lithium‐ion battery fires. By employing a nano‐CaCO_3_/GDL system as a thermally responsive crosslinker, the formulation maintains low viscosity and excellent foamability at ambient temperature. Upon exposure to the high heat of a battery fire, accelerated GDL hydrolysis rapidly releases Ca^2+^ from nano‐CaCO_3_, triggering rapid ionic crosslinking of sodium alginate. This transforms the aqueous foam into a stable, adhesive, solid‐like gel foam that completely encapsulates the battery. Such in situ gelation significantly enhances the foam yield stress, stability, and adhesion, enabling complete and durable encapsulation of battery surfaces, efficient long‐term cooling, and reliable heat isolation. Systematic characterization and full‐scale battery fire tests demonstrate that, compared with conventional AFFFs, thermally triggered gel foam demonstrates superior cooling performance for lithium‐ion batteries, primarily because of its higher thermal conductivity at equivalent ERs. It extinguishes single‐cell fires rapidly and strongly suppresses TRP in multicell modules while drastically reducing peak heat radiation, surface temperature, and toxic gas concentrations. The heat dissipation rate of the gel foam exceeds 1000 W, substantially higher than that of traditional water‐based agents (300–600 W), which is attributed to the synergistic effects of accelerated foam coverage, improved surface adhesion, and strengthened heat extraction enabled by elevated ERs. This work presents a promising and versatile approach for addressing the critical safety challenge of lithium‐ion battery thermal runaway. The stimulus‐responsive design principle opens new avenues for developing advanced firefighting agents tailored for next‐generation energy storage systems.

## Author Contributions

**Xiaoyang Yu**: conceptualization, methodology, writing – original draft. **Cuiping Li**: software, conceptualization. **Mingjun Xu**: project administration, resources. **Man Pun Wan**: review & editing, resources.

## Conflicts of Interest

The authors declare no conflicts of interest.

## Supporting information




**Supporting File**: advs77882‐sup‐0001‐SuppMat.docx.

## Data Availability

The data that support the findings of this study are available from the corresponding author upon reasonable request.
