# Supplementary material for: In Situ Generation of Gel Foam via Heat‐Accelerated Calcium Ion Release From Nano‐CaCO3 for Lithium‐Ion Battery Fire Suppression and Prevention
Source: Adv Sci (Weinh). 2026 Sep 27:e77882. Online ahead of print. doi: 10.1002/advs.77882 (PMC13616368; doi:10.1002/advs.77882)
Supplement: Supplementary file 1 [file ADVS-9999-e77882-s001.docx]

**Supplementary Material**

In situ generation of gel foam via heat-accelerated calcium ion release from nano-CaCO_3_ for lithium-ion battery fire suppression and prevention

Xiaoyang Yu^[a]^, Cuiping Li^[a]^, Mingjun Xu*^[a,b]^, Man Pun Wan^[b]^

[a]	State Key Laboratory of Fire Science
University of Science and Technology of China
Hefei 230026, China

[b]	School of Mechanical and Aerospace Engineering
Nanyang Technological University
Singapore 639798, Singapore
E-mail: mjxu@ustc.edu.cn; mingjun.xu@ntu.edu.sg (Mingjun Xu)

**S1 Materials**

SA (20–50 kDa), nano-CaCO_3_ (>98%), nano-CaSO_4_ (>99%), CaCl_2_ (>99%), and GDL (99%) were purchased from Macklin. Surfactant alkyl polyglycoside (APG, 50%) was purchased from Yousuo Company. Commercial foam extinguishing agents, namely, 3% aqueous film-forming foam (AFFF), 1% Class A foam, and 6% protein foam, perfluorohexanone (PFH), were supplied from Suolong Fire Protection Co., Ltd.

**S2 Preparation methods of SA gel foam**

SA was dissolved and combined with GDL and CaCO_3_ (a Ca^2+^ source to initiate gelation). When CaCO_3_ was used, a molar ratio of CaCO_3_ to GDL of 0.5 was consistently used to achieve near-neutral conditions. For all the formulations, a calcium-to-carboxyl molar ratio of 0.18 was defined as the 1× reference, where the carboxyl groups were contributed solely by SA. The crosslinking concentration was adjusted by multiplying this ratio, such as 0.5× (calcium-to-carboxyl molar ratio=0.09), 2× (calcium-to-carboxyl molar ratio=0.36), etc. After the SA and GDL solutions were mixed with the CaCO_3_ suspension, 1 wt% of the nonionic surfactant APG was added. Control groups were prepared by mixing SA/APG/GDL with CaCl_2_ or CaSO_4_. Foam used in the laboratory is produced by the modified Tessari method (Fig. S2). This modified technique utilizes two syringes connected by flexible tubing 30 mL medical syringe (HongDa Company) containing the foaming solution and another containing air. High shear rates required for effective gas-liquid mixing were achieved through multiple reciprocating cycles (push-pull motions) between the syringes [1]. The solution-air mixture at the syringe outlet rapidly fragmented into microbubbles.

**S3 Characterization methods of SA gel and gel foam**

The gelation rate was characterized by the gelation time, which was defined as the duration required for the mixture to solidify to the extent that it no longer flowed when the vial was inverted. For gelation time tests conducted at higher temperatures, the SA solution was equilibrated at the target temperature for at least 2 hours before Ca^2+^ was added.

The foaming ability was measured using the Ross-Miles method in this study. In the experiments, a 200 mL foaming solution was streamed through a capillary (the pipette outlet tube, ~2 mm in diameter) from a height of 1 m into a glass reservoir containing 50 mL of the same solution. Upon impact with the liquid surface, air entrainment generated foam, which continued to rise until the liquid flow ceased. The initial foam height was recorded to characterize the foaming ability. For the bubble size observation, the fresh foam was placed in a glass container (5 cm in diameter and 2 cm in height). The changes in bubble size over time at room temperature were observed and recorded via a microscope. ImageJ software was used to determine the area of each bubble in the images, allowing for the calculation of the diameter for each bubble (with a minimum of 150 bubbles analyzed per experiment). For the liquid drainage rate tests, we placed fresh foam into the liquid drainage apparatus reported in the previous work [2]. As the liquid drained from the foam into the beaker on the balance, the computer recorded the change in the mass of the drained liquid. The liquid drainage fraction at a certain time, representing the ratio of the mass of drained liquid to the initial foam mass, was recorded.

The microstructure of the lyophilised gel foam was examined using a ZEISS Sigma 300 scanning electron microscope. The thermal conductivity of the liquid foam was measured using a Hot Disk TPS 2500S (Hot Disk AB). FTIR spectra were acquired on a Thermo Fisher Scientific Nicolet iS20 spectrometer. The Micro-CT test of the freeze-dried foam was conducted on the Nano Voxel 3000 (Sanying Precision Instruments Co. Ltd). X-ray photoelectron spectroscopy (XPS) was performed on an ESCALAB 250Xi X-ray photoelectron spectrometer (Thermo Scientific, America) with Al Kα as the excitation source (hν = 1486.6 eV). The rheological properties of the gel foam were measured with a Discovery HR-10 rheometer. Rheological characterization involved both shear and oscillatory tests. In the shear test, the time-dependent viscosity of the foam was measured at a fixed shear rate of 100 s^-1^. Oscillatory tests were conducted with a 25 mm parallel-plate geometry (initial gap: 2 mm). Additionally, for each formulation, after the foam had stabilised for 1 h, amplitude sweeps were performed at a fixed angular frequency of 2 Hz, and frequency sweeps were conducted at a fixed strain of 1%. The intersection point of the loss modulus (G′′) and the storage modulus (G′) in the amplitude sweep test is recorded as the yield stress of the foam.

**S4 Lithium battery characterisation**

NCM ternary lithium-ion batteries (LIBs) without a safety valve were used, with a capacity of 73 Ah, dimensions of 220×140×10 mm, a nominal voltage of 3.6 V, and a weight of 804 ± 5 g. The onset temperature of self-heating (T_onset_) and the thermal runaway trigger temperature (T_trigger_) of the batteries were determined with a BTC-500 accelerating rate calorimeter (ARC, Fig. S11) from HEL (UK). Notably, T_onset_ denotes the temperature at which, under adiabatic conditions, the heat generation rate from internal exothermic reactions exceeds the heat dissipation rate to the surroundings. Moreover, T_trigger_ denotes the critical temperature under adiabatic conditions at which a cascade of internal exothermic chain reactions is fully activated, leading to an extremely rapid temperature increase and resulting in ignition or explosion.

Once the ARC test began, the battery was maintained in an adiabatic environment inside the ARC chamber, with no heat exchange with the surroundings. The ARC applied a "stepwise heating" method to thermally stimulate the battery. At each temperature step, after the battery and ambient temperatures were fully equilibrated and stabilized, the self-heating rate (SHR) of the battery was monitored. A SHR≥0.02 °C/min can be identified as the self-heating onset of the LIBs, while a SHR≥10 °C/s sustained for over 3 s can be determined as the thermal runaway triggering of the battery. If the SHR was≤0.02°C/min, it was concluded that no exothermic reaction had occurred within the battery, and the test proceeded to the next heating step. Once the SHR exceeded 0.02°C/min, it was determined that an exothermic reaction had initiated inside the battery. Thereafter, it began to follow the battery temperature changes, providing only an adiabatic environment without supplying additional heating.

The heat release rate (HRR) of the LIBs was examined primarily under a conical exhaust hood constructed in accordance with ISO 9705-1: 2016, for which a method and variants have been reported previously [3]. In summary, combustion products were collected by a conical hood and directed into the exhaust system (Fig. S12). An integrated measurement system and gas probes installed in the exhaust duct were employed to monitor the concentrations of O_2_, CO, and CO_2_ in the combustion products. The HRR of the battery was then calculated on the basis of the oxygen consumption principle. The ACR data are shown in Fig. S13-S15. With respect to the LIBs in this work (state of charge, SOC=100%), T_onset_ is 124.03 °C (Fig. S14), T_trigger_ is 199.48 °C (Fig. S14), and the total heat release is 2.0 MJ (Fig. S16).

**S5 Lithium battery fire extinguishing test**

A fire suppression test for lithium-ion batteries (CETC Lantian Technology Co., Ltd.) was conducted in an energy storage cabin (7.0×3.5×2.0 m, Fig. S10). To simulate the most hazardous scenario, the battery was charged to 100 % SOC. Thermal runaway was induced using a custom 600 W heating plate with the same dimensions as those of the battery [4-6]. Once thermal runaway was triggered, the heating plate was immediately turned off to minimize the impact of continued heating on the evaluation of the foam's cooling performance. Three thermocouple trees (TR#1~#3; 15 thermocouples) were used to measure the temperature above and on both sides of the battery (Fig. S10). The temperature was measured using a K-type thermocouple with a bead diameter of 0.5 ± 0.05 mm. A digital camera (Sony AX-600, 25 fps, 1920×1080 pixels) was placed outside the cabin to capture flame images. Gas sensors (CO_2_, CO, H_2_, H_2_S, CH_4_, and C_2_H_6_) were custom-made and waterproofed to prevent water ingress during fire suppression and were supplied by Honegeagle Co., Ltd. (China). Gases released during fire suppression were collected at personnel height (*h*=1.7 m). The fire suppression system was remotely and manually activated to discharge the extinguishing agent immediately upon visual detection of open flames. To evaluate the effectiveness of the foam, single-battery fire-fighting tests and thermal runaway propagation (TRP) inhibition tests involving a 9-cell battery array were performed. Unless otherwise noted, the foam flow rate was 5 L/min/m^2^. Comparative experiments were also conducted using water spray, water mist, and commercial fire-fighting foam (AFFF, Class A foam, and protein foam), separately, with a flow rate of 5 L/min/m^2^. The foam expansion ratio (ER) is defined as the volume ratio of gas to liquid within the foam, and the ER was fixed at 7.0 by adjusting the liquid and gas flow rates unless otherwise noted.

Fig. S1 Dynamic surface tension of the foaming solution with and without APG.

Fig. S2 The modified Tessari method used for making foam in the laboratory reported in the previous work [1].

Fig. S3 Comparison of foaming ability among 1 wt% SA/CaCO_3_/GDL, 1 wt% SA/CaCO_3_ (2×), and AFFF.

Fig. S4 EDS mapping of the freeze-dried foam made from 1 wt% SA/CaCO_3_/GDL (2×).

Fig. S5 The distribution of elements on the surface of freeze-dried foam made from 1 wt% SA/CaCO_3_/GDL (2×).

Fig. S6 Images of bubbles at different times in foam made from 1 wt% SA/CaCO_3_/GDL and SA/CaCO_3_ foam (2×), respectively.

Fig. S7 The volume of liquid drained from the foam of 1 wt% SA/CaCO_3_/GDL and SA/CaCO_3_ (2×) as a function of time.

Fig. S8 Micro-CT analysis of freeze-dried foams from SA/CaCO_3_ foam (2×) without GDL.

Fig. S9 Micro-CT analysis of freeze-dried foams from SA/CaCO_3_/GDL gel foams (2×).

Fig. S10 Setup for lithium battery firefighting and the TRP inhibition test.

|  |    |
| --- | --- |

Fig. S11 The image of accelerating calorimeter and NCM ternary LIBs.

Fig. S12 The image of a large-scale oxygen consumption calorimeter based on the ISO 9705-2016 standard.

Fig. S13 The variation of cell temperature, voltage and resistance of the battery over time in the ARC tests. The peak thermal runaway temperature reached 567.14 °C.

Fig. S14 Temperature rise rate *dT*/*dt* of the battery versus sample internal temperature in the ARC tests. The self-heating onset temperature was 124.03 °C, and the thermal runaway trigger temperature was 199.48 °C.

Fig. S15 Battery surface temperature versus time in the ARC tests. SOC=100%.

Fig. S16 The heat release rate (HRR) and the total heat release (THR) generated during thermal runaway of the battery. The THR during thermal runaway is about 2.0 MJ. The specific heat capacity of the battery *q_c_*=battery mass×ΔT/total heat generation during thermal runaway=1.05 kJ/kg/°C.

Fig. S17 Temperature distribution on the surface of the battery during the (a) single-cell fire-fighting test and (b) TRP test of a 9-cell module.

Fig. S18 Comparison of average heat dissipation rate under different Ca concentrations.

Fig. S19 Comparison of average heat dissipation rate under different ERs.

Fig. S20 Comparison of average heat dissipation rate across various fire extinguishing agents.

Fig. S21 Influence of ERs on the thermal conductivity of commercial AFFF and SA/CaCO_3_/GDL gel foam.

**References**

[1] X. Yu, H. Li, N. Kang, S. Lu, M. Xu, and M. P. Wan, Superelastic fire-safe aerogel with hierarchical structures via dual templates aided by microbubble engineering, Advanced Science 34 (2025) e06808.

[2] H. Li , X.Y. Yu , Z.H. Fu , S.X. Lu, Stabilization mechanisms of foams enhanced by xanthan gum and sodium carboxymethyl cellulose: Rheology–bubble structure interplay and predictive criteria for drainage delays, Carbohydrate Polymers, 366 (2025) 123901.

[3] ISO 9705-1:2016 Reaction to fire tests-Room corner test for wall and ceiling lining products Part 1: Test method for a small room configuration.

[4] X.T. Ren, D.P. Kong, P. Ping, X.J. Zhao, W. Gao, Y. Zhang, C. Yang, Z.K. Feng, D. Wang, Experimental study on the evolution patterns of thermal runaway and safety boundaries of commercial lithium-ion batteries under overheating failure, Materials Today Energy, 52 (2025) 101937.

[5] Y. Sun, X.K. Chen, H.B. Wang, C.S. Xu, X.N. Feng, Y.N. Zhang, J. Deng, M.G. Ouyang, Quantification the expansion behavior and deformation dynamic variation of lithium-ion batteries during thermal runaway, Energy, 348 (2026) 140253.

[6] M. Bruno, C. Francia, C. Baldo, S. Fiore, Selective recovery of lithium carbonate from lithium-ion battery black mass through microwave-assisted carbothermal reduction and water leaching, Journal of Environmental Management, 393 (2025) 127266.
